# “For me, it's just a piece of freedom”—Increased empowerment through physical activity promotion among socially disadvantaged women

**DOI:** 10.3389/fpubh.2022.867626

**Published:** 2022-07-27

**Authors:** Alexandra Sauter, Annika Herbert-Maul, Karim Abu-Omar, Ansgar Thiel, Heiko Ziemainz, Annika Frahsa, Stephanie Linder, Anne Herrmann-Johns

**Affiliations:** ^1^Department of Epidemiology and Preventive Medicine, Medical Sociology, University of Regensburg, Regensburg, Germany; ^2^Department of Sport Science and Sport, Friedrich-Alexander-University Erlangen-Nuremberg, Erlangen, Germany; ^3^Institute of Sports Science, Eberhard Karls University of Tübingen, Tübingen, Germany; ^4^Institute of Social and Preventive Medicine, University of Bern, Bern, Switzerland

**Keywords:** physical activity, empowerment, community-based participatory research, women's health, low socioeconomic status, ethnic minority, qualitative research, health promotion

## Abstract

**Introduction:**

Community-based participatory research (CBPR) is an effective health promotion approach for reaching socially disadvantaged groups. However, there is limited evidence on how such interventions and their effects can be reproduced across time and place. The present study examines the effects of BIG (i.e., movement as an investment in health), a long-standing German CBPR project. Since 2005, BIG has aimed to empower women in difficult life situations to increase control over their health determinants and reduce social inequalities by promoting physical activity. One of BIG's key features is its implementation in several German municipalities since 2005. This study explores (a) whether participation could change women's empowerment, and (b) how increased empowerment affects other areas of women's lives.

**Methods:**

With a total of 63 interviewees (i.e., 40 participating women, 7 trainers, 3 project coordinators, and 13 stakeholders), we conducted 53 semi-structured qualitative interviews in five BIG communities between 2007 and 2011. Some interviews were conducted with two people simultaneously. The interview guide contained questions on various dimensions of empowerment (e.g., project engagement, increased self-efficacy, and developed competencies). Framework analysis was used for the analytical process.

**Results:**

BIG contributed to women's empowerment in various ways, including increased self-efficacy, social network promotion, competency development, and increased motivation to change physical activity behavior. Women who took on added tasks and became more involved in project planning also strengthened their organizational empowerment. Furthermore, increased empowerment had a positive influence on the women's quality of life, family, and professional lives.

**Conclusion:**

The novel findings helped in understanding the effects of a complex empowerment-based approach that promoted physical activity among women in difficult life situations. Future research should focus on the long-term effects of these programs and their transferability to other sites. Further effort is necessary in the area of public health policy.

## Introduction

Regular physical activity (PA) is vital for maintaining health and wellbeing. It reduces the risk of overweight and obesity (1); has positive effects on the musculoskeletal system, mental health, cardiovascular, and metabolic systems; and has a preventive effect against some cancers (2, 3). The WHO recommends that adults engage in at least 150 min of moderate-intensity PA per week (2).

However, women with low socioeconomic status (SES) (commonly measured by education, income, and occupation) face barriers that hinder them from sustainably implementing these recommendations in their daily lives (4). In developed countries, regular PA follows a socioeconomic gradient, whereby women with higher education or income are likely to be regularly physically active than those who are more disadvantaged (5, 6). For example, in Germany, national survey data showed that 48.9% of women with low SES are physically inactive (including sports), compared to 18.9% of women with high SES (7). Furthermore, German data also showed that women are less physically active than men and that women with a migration background are significantly less physically active than women without a migration background (8). Various barriers hinder socially disadvantaged women from participating in PA offerings: (a) on an individual level, for instance, lack of knowledge of PA benefits and offerings, and language competencies; (b) on a social level, for example, lack of support in becoming physically active, religious cultural norms that hinder participation in PA offerings, family duties including childcare; and (c) on an environmental level, barriers such as lack of nearby locations and low walkability to PA offerings (4, 9–12).

Supporting socially disadvantaged women to become more physically active can therefore be seen as an important public health goal, as physical inactivity is one of the biggest public health problems of the twenty-first century (13). Thus, the development of need-based, low-threshold programs that address all three barrier levels described above is essential for reaching this particularly vulnerable group. Such programs are best accepted when the target group becomes involved in the planning and implementation of programs that consider their values and practices in sustainably engaging them in PA (14). Therefore, participation, and empowerment are two effective concepts for achieving social equity among people in difficult life situations (15, 16). Participatory approaches focus on equal cooperation between professionals and citizens in sharing knowledge, and experiences, thereby building capacities for collectively achieving common goals (17). Participation and empowerment are strongly linked. Participation is an essential concept for strengthening people's ability to act upon their own health. Empowerment is usually understood as a multilevel social process through which (disadvantaged) individuals, organizations, and communities work together to change their social and political environments to determine events that negatively influence their lives and health (18). There are three levels of empowerment in the literature that are intimately linked: individual (or psychological), organizational, and community empowerments (18, 19).

Several studies have shown that participatory interventions have a positive impact on health behaviors, health consequences, self-efficacy, and perceived social support outcomes across various conditions (20, 21). However, there is limited evidence on how such interventions and their effects can be reproduced across time and place (20). Thus, there is a special need to investigate the potential broader influence of participatory research through longitudinal study designs to better understand the value and transferability of highly context-specific health promotion projects and their effects on different sites (22, 23).

In this study, we examined the effects of the BIG (*Bewegung als Investition in Gesundheit*, i.e., movement as an investment in health) project, a long-standing German community-based participatory research (CBPR) project (24). Using a participatory approach, BIG aims to empower women in difficult life situations to increase control over the determinants of their health and reduce social inequalities (25). One of the key features of BIG is that it has been implemented in several municipalities in Germany since 2005. All municipalities used the same approach, which allowed for comparing processes and outcomes at the individual sites. Therefore, a large qualitative dataset was collected, making it possible to gain insights into how an empowerment-based participatory project can expand across communities and what its effects are. Previous work from Rütten et al. (25) and Röger et al. (26), have examined the effects of the BIG approach on women's individual, organizational and community empowerment using data from the first BIG community (25, 26). This study builds on existing evidence from these and other previous works. It employing a more comprehensive dataset from more BIG communities in rural and urban areas with different population sizes. To answer the research question, theoretical empowerment approaches used in previous BIG-studies have been employed (25, 26). Thus, this study will update and extend previous research on BIG and provide new in-depth insights into how to increase women's empowerment using a participatory approach designed to improve health and health behavior and reduce social inequalities.

*The study explored women's views on the following:*

- Whether participation in the BIG project could improve women's empowerment and
- How may increased empowerment affect other areas of women's lives?

## Methods

### The data set: The BIG project

The BIG project was originally developed in 2005 by the Department of Sport Science and Sport, Friedrich-Alexander-Universität Erlangen-Nuremberg, Germany (FAU) (27). The aim of the BIG project was to promote PA among women in difficult life situations (e.g., having a low household income, having a migration background, being unemployed, relying on welfare aid, or being a single mother) using community-based participatory research methods (28). Women actively participated in the planning and implementation of low-threshold exercise classes (e.g., free of charge, complementary childcare, proximity to place of residence). BIG was built upon the “cooperative planning approach” (29, 30), which equally involved women, researchers, and community-level policy and practice stakeholders (e.g., mayors, sports club representatives, and trainers) in the planning of PA offerings. Since all members of the cooperative planning provided specific resources (e.g., funds, access to sport facilities, contact information for the target women), it was possible to implement these programs at the community level. Cooperative planning encouraged women to express their interests and needs regarding PA offerings, thus empowering them to take control of their own health while gaining self-efficacy (26, 31). Since 2005, BIG has spread to 19 sites in Germany. Of these, four sites are currently starting the project. In seven communities, the project has been running for several years, and in nine communities, BIG was implemented (for about 4 years); however, the project could not be sustained. In 2019, across all sites, ~800 women regularly took part in about 60 different exercise classes (27).

The present study was conducted as part of a federally funded follow-up for the BIG project, termed NU-BIG, with the goal of assessing the long-term effects of BIG projects across all sites. The detailed study design of the NU-BIG is described elsewhere (24).

### Design

For the purpose of this study, all available qualitative data from 2005 to 2011 were pooled. For the comprehensive data analysis, we used 53 interview transcripts from five BIG communities [including interviews from pilot community A, see also Rütten et al. (25) and Röger et al. (26)], with a total of 63 interviewees (see Table 1). Interviews were initially conducted for process or outcome evaluation of the various BIG communities. Original data were generated between 2007 and 2011, usually 1–2 years after implementation of the project had started. Interviews were conducted with: (a) women who participated in BIG classes only (*n* = 18 interviews with 27 women; two group interviews); (b) women who participated in BIG classes and also joined the cooperative planning meetings (*n* = 12 interviews with 13 women; one group interview); (c) trainers of BIG classes (*n* = 7); (d) project coordinators who were responsible for planning and promoting BIG classes (*n* = 3); and (e) stakeholders who joined the cooperative planning meetings and/or helped in implementing the BIG program (*n* = 13). All interviews were held at comfortable places for the interviewees (e.g., café, mosque, research offices).

**Table 1:** Overview of interview data.

	**Community A**	**Community B**	**Community C**	**Community D**	**Community E**
Project term	Since 2005	Since 2008	2008–2017	2008–2017	Since 2011
Status	Ongoing	Ongoing	Discontinued	Discontinued	Ongoing
Community size (inhabitants)	112.385	152.270	117.000	72.137	74.048
**Interviewees**					
Women participating in BIG classes only	*n* = 10	*n* = 3	*n* =1 (group interview with nine women)	*n* = 3	*n* = 1 (group interview with two women).
Women additionally participating in cooperative planning meetings	*n* = 7	*n* = 3	–	–	*n* = 2 (one group interview with two women and one single interview).
Trainers	–	*n* = 2	*n* = 2	*n* = 3 (one male)	–
Project coordinators	–	–	*n* = 1	*n* = 1	*n* = 1
Stakeholders	–	*n* = 7 (one male)	*n* = 3 (two males)	*n* = 3 (two males)	–
Date of interviews	2007	2009	2010	2010	2011

Interviews were conducted by research assistants from the Department of Sport Science and Sport of Friedrich-Alexander-Universität Erlangen-Nuremberg, who also provided scientific support for the implementation of BIG at individual project sites. All interviews were audiotaped and transcribed verbatim. The interview guides consisted of eight fixed sets of questions, which considered the following concepts from health promotion in particular. First, social capital, defined as (a) an individual's membership of a social group (relational aspect) and (b) the benefits an individual derived from the social network (material aspect) (32, 33). Second, self-efficacy, defined as an individual's belief in their own capacity to control certain behaviors (34). The eight sets of questions are: (a) relevance of BIG, (b) motivation to participate, (c) sports biography, (d) comparable women-only offerings in the hometown, (e) organization and low-threshold nature of the classes offered, (f) initiated behavioral changes, (g) relationship between participating women, (h) suggestions for program adaption.

### Ethical considerations

The Ethics Committee of the Friedrich-Alexander University Erlangen-Nuremberg granted ethical approval for this follow-up study (approval number: 247_20 B). All interviewees gave informed consent for the interview, the audio recording, and the scientific use of their accounts. All transcripts were anonymized so that no inferences could be drawn about the interviewees or others mentioned in the interview.

### Analytical process

Interview data were analyzed using the framework method (35). This approach provides a systematic model for mapping and interpreting qualitative data. Thus, it was considered appropriate for developing an insightful, in-depth understanding of women's experiences within BIG and the project's impacts on their daily lives (36). The analysis consisted of five systematic and interconnected steps.

Step 1—Familiarization: This was done on the interview data by rereading the narratives several times, which was particularly important, as coders were not involved in the data collection process.

Step 2—Coding: We followed a combined deductive–inductive coding strategy. For this strategy, codes were based on the empowerment theory of Rappaport (18), Zimmerman (19) and Laverack (37), using the three domains of empowerment (individual, organizational, and community), as described in Table 2. The first author (A.S.) coded three interviews with several codes for each level of empowerment (e.g., organizational empowerment used the following codes: “participation in cooperative planning meeting,” “equal say,” and “advocate matters of personal importance”). To ensure reliability, the generated codes were discussed with a second coder (a master's student, experienced in analyzing qualitative data) until consensus was reached, while continually returning to the coded quotes to check for meaning and context, as recommended by Pope et al. (38). In the second step, the transcripts were coded inductively by the first author, with emphasis on newly occurring phenomena that were not included in the established levels of empowerment, but which were essential in answering the research questions.

**Table 2:** Framework showing themes based on empowerment theory.

**Level of empowerment**	**Description**	**Interview guide question**	**Themes**
Individual	Process of change with intrapersonal, interactional and behavioral components and which is related to increased self-efficacy, perceived competencies or skill development (18, 55).	• Have you experienced any changes as a result of participating in the BIG project? • How has the BIG project affected your everyday life? How would you describe your everyday life before BIG and now with (or after) BIG? • Do you now have more knowledge about your capabilities to be physically active? • Do you also participate in sports apart from BIG offerings?	• Feeling more confident and gaining self-efficacy through class participation facilitate women's PA. • Receiving fellowship and social support within the BIG community. • Joining planning processes helps strengthen personal competencies and skills. • Class enjoyment and improved PA foster motivation to set and attain new goals and implement further health-related behaviors.
Organizational	Processes and structures that enhance members' skills and provide them with the mutual support necessary to effect community level changes. Fosters individuals to improve organizational effectiveness by effectively competing for resources, networking with other organizations, or expanding influence (19, 55).	• Was there an exchange of experience/knowledge with other participants? How would you personally rate this exchange? • Did any new opportunities arise for you as a result of attending the cooperative planning meetings? • To what extent were you able to contribute your own ideas to the cooperative planning meetings/the BIG classes?	Low-level participation and shared decision making can foster women's involvement.
Community	Links interactions between individuals and organizations working together in an organized fashion to improve local living conditions and thereby initiate changes in a larger social system (37, 55).	With which BIG partners did you have contact (members of local organizations, exercise instructors, women from the target group)?	Collective problem assessment helps to create “safe spaces” to practice PA.

Step 3—Constructing the framework: The set of codes was discussed with the research group (A.H-M., K.A-O., A.T., H.Z., S.L., A.H-J.) to form a working analytical framework that was best suitable for addressing the research questions in a meaningful way.

Step 4—Applying the analytical framework: All transcripts were coded by the two coders using the analytical framework. Step 5—Charting and interpreting data: Similar codes were combined to develop themes. All developed themes and the relationships between them were reviewed by members of the research team to check whether the themes were coherent and captured the most relevant data features.

The analytical process was conducted in German. Only the quotations used in this paper were translated into English. The translation was checked by several authors for meaning and content to ensure that no information was lost during the translation process.

## Results

### Individual empowerment

Feeling more confident and gaining self-efficacy through class participation facilitate women's PA.

One of the main findings across all communities was that the women experienced increased self-efficacy. The majority of women who regularly participated in the classes highlighted that they soon underwent a personal change by experiencing progress in their own PA abilities, especially women in swimming classes. After a few sessions, the women felt more confident while exercising and began to believe in their own abilities. Through increased self-efficacy, the women felt proud and empowered to set new goals and continue the training, for example, getting a swimming certificate, trying new sports classes, or swimming in the open sea.

“*Before [BIG], I was so jealous that my children could swim, and I just sat on the beach [...]. And now I have achieved this. I am so proud of myself that I have learned how to swim.”* (Woman, Community A).

“*[After a while] the women in the swimming class were brave enough to go into deeper water. In the first sessions, I had to hold them by their swimming pants. Later, they dared to swim back and forth without any assistance. When they get to this point, they are really motivated. The swimming teacher thinks about training them to get a swimming certificate.”* (Project Coordinator, Community E).

However, according to the professionals involved in the project, increased self-efficacy for some women seemed to be limited to their own sporting activities within BIG classes. Additional training to become an intercultural sports assistant was only taken up by some women but rejected by others. Low confidence was identified by the interviewed professionals as the reason for the women's rejection of completing this additional training and perhaps running courses themselves in the future. Also, a lack of incentives to invest time and energy in this further training were named as additional barriers.

“*When I suggested this [training as an intercultural sports assistant] to them, they looked at me in surprise. The first thing they wanted to say was, “Are you at the right address?” When I tried to convince them that they could actually do it, they were very thoughtful and went home. The next day or a week later, they said, “Well, we can't do it.””* (Expert, Community D).

Receiving fellowship and social support within the BIG community helps to continue PA classes and foster mental wellbeing.

Another dimension of empowerment addressed by the BIG project in all communities was the experience of community and social support. In particular, unemployed, or widowed women who had little contact with others valued the possibility of getting in touch with other women in the classes. They described these contacts as enrichment for their everyday lives and as a way out of their social isolation at home. Belonging to a community strengthened these women's feelings of importance and self-worth, as they were now heard and seen by others and had the opportunity to bond with others. The BIG community was considered important not only to motivate each other to participate in PAs but also to have a place to talk about everyday problems and topics of importance to women.

“*[At the end of] most BIG classes, there is still time to talk and motivate each other. That is very important to them [women]. In the meantime, problems are discussed after the classes. That's why the women like coming there so much.”* (Trainer, Community C).

“*Some of our participants are widowed. They no longer have many opportunities to be sociable or to go out. BIG helps them overcome this hole by having a reason to leave the house.”* (Trainer, Community D).

H The participating women obtained benefits through their membership in the BIG project because the women supported each other. Participating mothers, for example, benefited from the childcare provided, which was often done by other women. This support allowed mothers to join BIG classes on a regular basis and take time out from their demanding everyday lives to do something for themselves.

“*Of course, it's been good for me, because it's an hour I have for myself. It's my first child, and you're like a supermom. You concentrate only on your child. Thus, it's a nice thing to say: I'm doing something for myself again. That is simply a piece of freedom for me.”* (Woman, Community E).

Furthermore, taking over childcare increased the childsitter's empowerment as well, as it gave them a meaningful task and helped some to find part-time employment. Thus, in several cases, BIG not only empowered the participating women but also benefited the women who contributed to the project.

“*I also wanted to join the swim class, but I'm a professional child nurse and they were looking for child care [for the swim class]. So, I took the job, because for me it's more important that my friends [with children] can join the class. And for me, it was a good opportunity to have a part-time job.”* (Woman, Community B).

However, women of Muslim faith in particular reported partnership conflicts because of their participation in BIG classes and lack of support from their husbands. Some women lost weight and gained more self-confidence due to their changed appearances. In some cases, husbands viewed their wives' new body image with skepticism and reservation. Although some women felt empowered by their new confidence in asserting their needs and activities, others reported feeling uncomfortable with these marital conflicts and decided to stop the BIG program in favor of a harmonious partnership.

“*Yes, the relationship with my husband [has changed]. In the past, I always did everything right for him. And now I can say, “Cook your own dinner, I'll be home later today.””* (Woman, Community A).

“*My family and husband come first. It's better than starting a fight just because of losing weight.”* (Woman, Community C).

Joining planning processes helps strengthen personal competencies and skills.

Interviewees who also participated in the cooperative planning group meetings reported gaining additional competences. Working together with different interest groups and managing the organizational work fostered the women's project management skills, social skills, and self-confidence.

“*I learned a lot about how to deal with different people, especially with the women in the BIG classes, the trainers, with you, the scientific staff and with all the different institutions involved. How to proceed in a cooperative planning process—quite simply, how to write emails, letters, and so on.”* (Woman, Community A).

Additionally, participating in BIG seemed to be a great way to improve the women's German language skills. As the project progressed, the most common change was the transition from being shy to becoming confident when talking to others in German. Interviewees valued the new opportunities to communicate their thoughts to other planning group members and to have a say. They felt empowered to become actively involved in the planning and implementation processes.

“*I felt very good whenever I came to this meeting [cooperative planning]. I could talk to all these people. I was able to express my ideas; they were heard, and they were taken into account. I participated, and it made me feel good.”* (Woman, Community A).

These new language competencies also empowered many women to lead their daily lives more independently (e.g., by handling administrative issues, such as going to public offices without a translator or applying for a job) and made it easier for them to (re)enter the labor market.

“*I haven't worked for 8–9 years because I stayed at home with my children […] I didn't feel comfortable talking to people. The BIG project helped me a lot. Now, I'm doing an internship twice a week. If it wasn't for BIG, I wouldn't be able to speak with people.”* (Woman, Community B).

Class enjoyment and improved PA foster motivation to set and attain new goals and implement further health-related behaviors.

Interviews with the women in the target group and the narratives of the trainers and coordinators demonstrated a change in the women's motivation and enjoyment in participating in the various classes. This change was described as regular and enthusiastic class participation. It was also conveyed in the women's booking of further classes the following semester, even when class fees could not be fully covered. The professionals explained this behavior by the positive changes the women experienced by attending classes (e.g., increased fitness, increased wellbeing, and body positivity). Furthermore, improved abilities, such as swimming skills, enabled the women (especially the Muslim women) to do joint activities with their children in the water. They also felt empowered to act if their children needed rescue in an emergency.

“*I often notice that the women continue booking [further classes], even if the course fee is no longer covered by the health insurance. Because they simply notice that it's good for them. Less pain, more mobility, and improved general fitness.”* (Trainer, Community C).

“*Of course, I would like to learn more [...] For example, other swimming styles. Diving is also important. If I am with my child at sea and he sinks, I have to learn how to rescue him.”* (Woman, Community A).

Interviewees also reported that the women integrated PA into their daily routines as the project progressed. Daily transportation was more often by bike or foot, and leisure time activities with friends or family were also increasingly linked with PA. For some women, this became an opportunity to find new joint hobbies with their partners.

“*Before [BIG] I have never done any sports at all. Now, I ride my bike or walk when I have to go somewhere […] I am not so averse to PA anymore.”* (Woman, Community A).

“*We [my husband and I] also do something together from time to time. I guess, if I wouldn't go to the BIG class now, he would have done his sport, and I would have watched or had no connection to him at all. But now we can do [exercises] together.”* (Woman, Community A).

A few women also reported that they felt motivated to change other health-related behaviors, such as their diet, due to participation in BIG. Furthermore, participation in the BIG project seemed to have positive effects on their general wellbeing and mental health. This was also reflected in their interactions with family members, who often noticed and commented on their wives' or mothers' changed state of mind.

“*For example, some had problems with depression, they have become more balanced [since BIG]. And of course, this has an effect on the family and also on the children […] some women told me, their husbands appreciate BIG because every time their wives come home after class, they are in a good mood.”* (Trainer, Community C).

### Organizational empowerment

Low-level participation and shared decision making can foster women's involvement.

Across all communities, interviewees valued the openly structured and interactive format of many classes. In this way, the women felt that their needs and wishes were being considered in every class, as they could always express their wishes and needs to the trainers.

“*The trainer is very responsive to us and we are able to express our wishes, what we would like to do [in the class]”* (Woman, Community D).

However, regarding involvement in the cooperative planning group meetings, women, stakeholders, and coordinators involved in the CBPR process reported several difficulties. In particular, in the beginning, when the women had lower language levels, uncertainties about expressing their views in front of other people, especially decision-makers (e.g., mayors), reduced some women's empowerment in becoming actively involved in the planning process. Furthermore, the format of the cooperative planning meetings, with its official character and the “academized” topics, were rated by some as unfamiliar and uninteresting for lay people. Many of the interviewed women were not averse to participating in planning processes or having a say in decisions. However, in most communities, informal formats, such as monthly breakfasts, made these women feel more comfortable raising their wishes and concerns and exchanging ideas with others. This was due to easier language speaking ability, flat hierarchies, and more familiar locations (e.g., district meetings and family centers).

“*The [cooperative planning] meetings were okay. I always took part, but I couldn't talk much because somehow the mayor was there and the head of the sports department. And you don't know what to say […] Because it could be that I just say words wrong, and then it sounds stupid. So I preferred to say nothing at all.”* (Woman, Community A).

“*In general, it was difficult to convince the women to go to the town hall and participate in the cooperative planning [...]; many women simply didn't want to come. They either don't have time, or it's an excuse, or they don't feel like dealing with it and planning something. It has proven to be easier to organize a women's breakfast event or a homogenous group meeting. Something that is more sociable.”* (Project Coordinator, Community E).

### Community empowerment

Collective problem assessment helps to create “safe spaces” to practice PA.

For women of the Muslim faith, the prospect of PA classes based on their needs helped activate their empowerment. They soon developed a high level of problem awareness and problem-solving skills to obtain access to public swimming pools and create class environments that suited their religious and cultural regulations. This included measures such as covering windows and doors in swimming pools to protect them from male glances and advocating special swimwear for Muslims in the swimming pools. In cooperation with different community organizations and initiatives, women-only indoor pool hours could be established in most of the participating communities. This served as an addition to the PA classes.

“*Other women from our Turkish community told us about it [the BIG project]. Together, we went to the city administration and explained to them that we also wanted to have a women-only indoor swimming class [in our neighborhood]. She gave us registration forms, which I filled out, and soon afterward, it started.”* (Woman, Community E).

## Discussion

### Principal findings

In this study, we examined the effects of BIG, a CBPR project, aimed at empowering women in difficult life situations through PA promotion to gain more control over their health. We analyzed qualitative interviews from five communities with participating women, project coordinators, and stakeholders using a multilevel empowerment framework (19, 39). An important finding from this study is that, within the BIG project, the transfer of this empowerment-based CBPR program from one community to another is possible, even though the transfer of such complex interventions is described as difficult (40). All five communities achieved initiated changes at all three levels of empowerment, with the greatest changes observed in individual empowerment. Our findings could confirm existing evidence from Rütten et al. (25) and Röger et al. (26). By using a larger set of data from different communities, we could show that some of the effects of the BIG project are not exclusive for pilot community A [see also Rütten et al. (25) and Röger et al. (26)], but could also be found when transferring the project to other sites. Women who also participated in the cooperative planning meetings had even greater effects on their individual and organizational empowerment, as they were able to contribute their ideas and thus influenced the course of the project. These women reported additional skill development, such as organizing meetings, improving their German language speaking skills, and interacting with agencies from other institutions. This skill development even helped some back into the workforce (25, 26). The results of our study also show that some women feel a certain ambiguity toward the BIG project. Although women were proud of their increased empowerment and the “safe” environment they built up together, for some women (especially those of the Muslim faith), increased self-confidence were viewed critically by their spouses. Consequently, some felt that they must decide whether to continue their participation in PA classes or quit in favor of a harmonious family life.

### Comparison with other studies

To our knowledge, the BIG project is the first CBPR project to use an empowerment approach to promote PA among disadvantaged women. Other studies used empowerment approaches mostly for other groups, other health promotion fields, or other intervention strategies different from the cooperative planning process, with most having an educational character (41–43). A few CBPR projects have identified several effects of empowerment at the individual level, such as greater confidence in an individual's abilities, acting as a role model for family members, receiving social support from peers, and becoming motivated to change certain health behaviors (44, 45). Nevertheless, CBPR projects using an empowerment approach face many challenges. In our study, for example, the women valued the opportunity to actively participate in the project. However, it often seemed challenging to convince women (especially those with migration backgrounds) to participate regularly in formally structured planning group meetings. Fröberg et al. (46), for example, showed that in their empowerment-based school intervention, the involved students had less interest in discussions and goal-setting strategies and could not be convinced of the relevance of an active everyday life to their later health. Consequently, their 2-year intervention study showed no positive effect on the student's sedentary behavior or PA. In the BIG project, this barrier was partially resolved by all communities through organizing more informal meetings for the involved women (e.g., women's breakfast sessions), with a low threshold, where project topics could be discussed easily.

Other approaches can be found in the literature on how to attract socially disadvantaged groups of people to participate in CBPR projects. For example, a Lebanese CBPR project with the aim of improving the reproductive and mental health of women in disadvantaged communities in Beirut established a local committee in which women in the target group had the opportunity to explain their living circumstances and express their needs (47). The research team attempted to listen to the women with time and care to build trust, which was seen as a major factor in successfully recruiting them for the project trial. Nevertheless, some women lost interest in participating in the committee. Out of 20 women, only six women maintained interest over the remaining 2 years. Still, the remaining women felt empowered and ownership of the study. Most women were unemployed for most of their lives. They saw in their participation a meaningful and productive use of their time.

Overall, with regard to engaging people from minority communities, it is valuable to find peer champions who feel empowered to publicly speak for their peer groups in stakeholder group meetings or project committees and to get others involved in the planning process. Israel and colleagues, for example, set up field offices in their CBPR project and hired local community members as staff who were similar to the project participants (e.g., culture, and language) (48). Staff positions could include field coordinator, interviewers, and intervention staff. This ensured that the target group could be addressed and involved in all steps of the project in a culturally sensitive way. Avery et al. (44) used a similar approach, employing lay health promoters as trust builders. Trust and interpersonal interaction are important facilitators of empowerment processes, especially for people with migration backgrounds. The lay health promoters had continuous dialogues with the community members and helped with participant recruitment and language interpretation. In particular, the participating women benefited from knowing their neighborhood lay health promoters, as they trusted that the promoters' recommendations (e.g., for PA activities) would be culturally sensitive.

While we did not explicitly include “trust” in our interview guide, it seemed that, especially for women of the Muslim faith, trust in a need-based workout space (e.g., no men present, and protected from others' views) was crucial for reaching these women and encouraged long-term participation. These results are consistent with the findings of a previous pilot study on Community A (49).

### Strengths and limitations

We conducted a secondary analysis of existing data (25, 26) which was supplemented and updated by additional interview data to answer the research question underpinning this current study. Due to this in-depth and rich data material shows how the provision of need-based PA offerings and the dynamics of interactions between the participants strengthen the women's individual empowerment in terms of self-efficacy, competencies, power, and social capital (e.g., advancing social networks, bonding with other women, experiencing group solidarity, and belongingness).

The comprehensive and rich dataset with interviews of involved women, trainers, coordinators, and planning group members from different project sites enables a triangulation of different perspectives. This has made it possible to obtain a comprehensive picture of the effects of the BIG project on women's empowerment and the broad effects on women's everyday lives. Given the flexible and emerging nature of CBPR projects, it is noteworthy that the BIG approach was transferable to all five communities and initiated changes in all three levels of empowerment at all sites. Despite this, effects at the organizational or community levels were less visible.

Although data collection for this study occurred several years ago, and situations at the various sites may have changed somewhat, the data under investigation helped in answering an essential research question by focusing on how to strengthen a vital aspect of health promotion. In addition, the participants invested time and effort in providing this data. We believe that it would be unethical not to use this information to increase the evidence base in this field, inform future research, and provide practical implications for health promotion.

However, the findings from this study may not simply be transferable to other countries because of specific German situations or cultural contexts. The evaluation of empowerment-based CBPR projects is also difficult. Furthermore, the operationalization and measurement of these concepts have proven to be challenging in health promotion research and practice, given that there are varying conceptualizations of empowerment and participation (50). Thus, we used a classical empowerment approach based on the concepts of Rappaport and Zimmermann to analyze our findings. Using other concepts of empowerment may highlight other aspects of the data.

### Implications for future research and practice

Our results suggest that when intending to foster empowerment processes through local PA interventions among socially disadvantaged women, establishing cooperative planning groups could be effective in involving women in planning and implementation processes. The findings of this study highlight that women's participation in such groups empowers them and gives them more self-confidence, which can help in various areas of their lives. However, our study also shows that not all women benefit equally from participating in cooperative planning groups. In particular, women with very low language levels may find it difficult to participate in these meetings. In addition, the participation of authority figures may pose barriers to participating actively or at all. For this reason, more informal formats, such as women's breakfasts, may also be suitable for this target group, where women may be able to talk more freely about topics that interest them and affect their lives. The results of such low-threshold meetings could then be integrated into the cooperative planning processes, for example, by the facilitating researchers. Further, including a peer champion to motivate others to join the planning process and who feels encouraged to speak for the peer group in the meetings might help overcome the challenge of engaging people from the target group in the project.

For some participating women, obtaining family support was challenging, as partners were skeptical about the program or viewed women's new self-confidence negatively. Including family-based intervention strategies might be a way to better convince spouses of the purpose and value of PA classes. Furthermore, it might be an avenue for supporting PA in the home environment and during leisure time with family members.

Using the concept of empowerment in a CBPR project is always accompanied by methodological difficulties, as there is a degree of uncertainty about how the concept should be understood and operationalized (50). Researchers may struggle due to the variety of possible indicators used to measure empowerment and to define whether empowerment is to be seen as a process or an outcome (51, 52). Some even argue that one cannot empower others. Instead, the target group must empower itself and define empowerment indicators (39, 53). Thus, for better clarity of project goals, to more actively involve the target group in the research process, and to increase their commitment to the project, the target group should be involved in preliminary methodological considerations (54). This would allow the target group to determine the domains that should be addressed later.

## Conclusion

We examined the effects of a CBPR project (BIG) designed to empower socially disadvantaged women through PA promotion at five different project sites. Interviews from five project communities showed that the BIG approach and some of its key effects were transferable to other sites. The findings highlight that empowerment can be reached on an individual level. According to the women's perceptions, participating in PA classes strengthened their self-efficacy and confidence in their own abilities, expanding their social networks and affecting their PA behaviors and those of their families. For women who also participated in the cooperative planning meetings, empowerment processes were also initiated on an organizational level, and project participation had a greater impact on their private lives, such as reentry into the workforce and greater self-reliance in everyday life. In sum, this study reveals novel findings that help us understand the effects of a complex empowerment-based approach that promotes PA among women in difficult life situations. Future research should focus on the long-term effects of empowerment-based CBPR programs and their transferability to other sites. This will also warrant further efforts in the area of public health policy, such as long-term funding for effect-proven research interventions on a broad scale. Furthermore, governments need to provide resources to create socio-political environments at a local level to strengthen community capacities in identifying health needs in their communities and to sustainably implement programs that foster the health behaviors of minority groups.

## Data availability statement

The data generated and analyzed in this study is available upon reasonable request from the corresponding author alexandra.sauter@ukr.de.

## Ethics statement

The studies involving human participants were reviewed and approved by Ethik Kommission der Friedrich-Alexander-Universität Erlangen-Nürnberg. The patients/participants provided their written informed consent to participate in this study.

## Author contributions

KA-O and HZ initiated the study. AS analyzed the data, designed the study, and drafted the manuscript. AH-J participated in writing the manuscript. AH-M supports the provision and processing of the data. AH-M, KA-O, HZ, AT, SL, and AH-J contributed to study conception, data analysis, and critically revised the manuscript. AF conducted interviews and critically revised the manuscript. All authors read and approved the final manuscript.

## Funding

This described project and the work on this manuscript was supported by the German Federal Ministry of Education and Research, Grant No. 01EL2012B and Grant No. 01EL2012A.

## Conflict of interest

The authors declare that the research was conducted in the absence of any commercial or financial relationships that could be construed as a potential conflict of interest.

## Publisher's note

All claims expressed in this article are solely those of the authors and do not necessarily represent those of their affiliated organizations, or those of the publisher, the editors and the reviewers. Any product that may be evaluated in this article, or claim that may be made by its manufacturer, is not guaranteed or endorsed by the publisher.

## References

[1] Warburton DE Nicol CW Bredin SS. Health benefits of physical activity: the evidence. CMAJ. (2006) 174:801–9. 10.1503/cmaj.051351 16534088 PMC1402378

[2] World Health Organization. Global Recommendations on Physical Activity for Health. Geneva: WHO (2010). 26180873

[3] Pfeifer K Banzer W Ferrari N Füzéki E Geidl W Graf C. Empfehlungen für Bewegung [Recommendations for physical activity]. In: Rütten K Pfeifer K editors. Nationale Empfehlungen für Bewegung und Bewegungsförderung [National recommendations for physical activity and physical activity promotion] Bundeszentrale für gesundheitliche Aufklärung. Sonderheft 3 (2017). p. 18–49. 10.1055/s-0042-123346 28399579

[4] Withall J Jago R Fox KR. Why some do but most don't. Barrieres and enablers to engage low-income groups on physical activity prgrammes: a mixed methods study. BMC Public Health. (2011) 11:507. 10.1186/1471-2458-11-507 21711514 PMC3141466

[5] Cerin E Leslie E. How socio-economic status contributes to participation in leisure-time physical activity. Soc Sci Med. (2008) 66:2596–609. 10.1016/j.socscimed.2008.02.012 18359137

[6] Ball K Carver A Downing K Jackson M O'Rourke K. Addressing the social determinants of inequities in physical activity and sedentary behaviours. Health Promot Int. (2015) 30(Suppl. 2):ii18–9. 10.1093/heapro/dav022 25855784

[7] Krug S Jordan S Mensink GB Muters S Finger J Lampert T. Physical activity: results of the german health interview and examination survey for adults (DEGS1). Bundesgesund Gesundheits Gesund. (2013) 56:765–71. 10.1007/s00103-012-1661-6 23703496

[8] Robert Koch Institut. Gesundheitliche Lage der Frauen in Deutschland. Gesundheitsberichterstattung des Bundes. Gemeinsam getragen von RKI und Destatis. RKI, editor. Berlin (2020).

[9] Frahsa A Streber A Wolff AR Rutten A. Capabilities for physical activity by turkish- and russian-speaking immigrants aged 65 years and older in Germany: a qualitative study. J Aging Phys Act. (2020) 2020:1–13. 10.1123/japa.2018-0446 31914420

[10] Calderwood C Minnen ME Phetmisy CN Kidwell KE French KA King DD. Understanding how family demands impair health behaviors in working sole mothers: the role of perceived control over leisure time. Appl Psychol Health Well Being. (2021) 14:362–82. 10.1111/aphw.12307 34491619

[11] Camhi SM Debordes-Jackson G Andrews J Wright J Lindsay AC Troped PJ. Socioecological factors associated with an urban exercise prescription program for under-resourced women: A mixed methods community-engaged research project. Int J Environ Res Public Health. (2021) 18:8726. 10.3390/ijerph18168726 34444473 PMC8394072

[12] Carver A Akram M Barnett A Mellecker R Cerin E. Socioeconomic status and physical activity among mothers of young children in an Asian city: the mediating role of household activities and domestic help. Int J Environ Res Public Health. (2020) 17:2498. 10.3390/ijerph17072498 32268519 PMC7178123

[13] Blair SN. Physical inactivity: the biggest public health problem of the 21st century. Brit J Sports Med. (2009) 43:1–3. 19136507

[14] Marent B Forster R Nowak P. Theorizing participation in health promotion: a literature review. Soc Theory Health. (2012) 10:188–207. 10.1057/sth.2012.2

[15] World Health Organization. Declaration of Alma Ata (International Conference on Primary Health Care). Copenhagen: WHO Europe (1978).

[16] World Health Organization. Ottawa-Charta for Health Promotion. Copenhagen: WHO Europe (1986).

[17] Minkler M Wallerstein N Wilson N. Improving health through community organization and community building. In: Glanz K Rimer B Viswanath K editors. Health Education and Health Education Theory, Research, Practice. San Francisco, CA: Jossey-Bass (2008). p. 37–58

[18] Rappaport J. Terms of empowerment/examples of prevention: toward a theory for community psychology. Am J Commun Psychol. (1987) 15:121–48. 10.1007/BF00919275 3604997

[19] Zimmerman M. Empowerment theory. In: Rappaport J Seidman E editors. Handbook of Community Psychology. Boston, MA: Springer (2000).

[20] O'Mara-Eves A Brunton G Oliver S Kavanagh J Jamal F Thomas J. The effectiveness of community engagement in public health interventions for disadvantaged groups: a meta-analysis. BMC Public Health. (2015) 15:129. 10.1186/s12889-015-1352-y 25885588 PMC4374501

[21] Haldane V Chuah FLH Srivastava A Singh SR Koh GCH Seng CK. Community participation in health services development, implementation, and evaluation: a systematic review of empowerment, health, community, and process outcomes. PLoS ONE. (2019) 14:e0216112. 10.1371/journal.pone.0216112 31075120 PMC6510456

[22] Wolfenden L Chai LK Jones J McFadyen T Hodder R Kingsland M. What happens once a program has been implemented? A call for research investigating strategies to enhance public health program sustainability. Aust N Z J Public Health. (2019) 43:3–4. 10.1111/1753-6405.12867 30690829

[23] Milat AJ King A Newson R Wolfenden L Rissel C Bauman A. Increasing the scale and adoption of population health interventions: Experiences and perspectives of policy makers, practitioners, and researches. Health Res Policy Syst. (2014) 12:1–11. 10.1186/1478-4505-12-18 24735455 PMC3996855

[24] Abu-Omar K Ziemainz H Loss J Laxy M Holle R Thiel A. The long-term public health impact of a community-based participatory research project for health promotion among socially disadvantaged women-a case study protocol. Front Public Health. (2021) 9:628630. 10.3389/fpubh.2021.628630 33912528 PMC8075296

[25] Rütten A Roger U Abu-Omar K Frahsa A. Empowerment of women in difficult life situations: the BIG project. Gesundheitswesen. (2008) 70:742–7. 10.1055/s-0028-1103262 19085670

[26] Röger U Rutten A Frahsa A Abu-Omar K Morgan A. Differences in individual empowerment outcomes of socially disadvantaged women: effects of mode of participation and structural changes in a physical activity promotion program. Int J Public Health. (2011) 56:465–73. 10.1007/s00038-010-0214-8 21076931

[27] Herbert-Maul A Abu-Omar K Frahsa A Streber A Reimers AK. Transferring a community-based participatory research project to promote physical activity among socially disadvantaged women-experiences from 15 years of big. Front Public Health. (2020) 8:571413. 10.3389/fpubh.2020.571413 33072709 PMC7542241

[28] Brush BL Mentz G Jensen M Jacobs B Saylor KM Rowe Z. Success in long-standing community-based participatory research (CBPR) partnerships: a scoping literature review. Health Educ Behav. (2020) 47:556–68. 10.1177/1090198119882989 31619072 PMC7160011

[29] Rutten A Gelius P. Building policy capacities: an interactive approach for linking knowledge to action in health promotion. Health Promot Int. (2014) 29:569–82. 10.1093/heapro/dat006 23468466

[30] Sommer R Linder S Ziemainz H Gelius P. Key performance indicators of cooperative planning processes: case study results from German sport science and physical activity promotion projects. German J Exerc Sport Res. (2021) 52:24–38. 10.1007/s12662-021-00745-3

[31] Frahsa A Rutten A Roeger U Abu-Omar K Schow D. Enabling the powerful? Participatory action research with local policymakers and professionals for physical activity promotion with women in difficult life situations. Health Promot Int. (2014) 29:171–84. 10.1093/heapro/das050 22987843

[32] Eriksson M. Social capital and health–implications for health promotion. Glob Health Action. (2011) 4:5611. 10.3402/gha.v4i0.5611 21311607 PMC3036711

[33] Hawe P Shiell A. Social capital and health promotion: a review. Soc Sci Med. (2000) 51:871–85. 10.1016/S0277-9536(00)00067-8 10972431

[34] Bandura A. Health promotion by social cognitive means. Health Educ Behav. (2004) 31:143–64. 10.1177/1090198104263660 15090118

[35] Gale NK Heath G Cameron E Rashid S Redwood S. Using the framework methods for the analysis of qualitative data in multi-disciplinary health research. BMC Med Res Methodol. (2013) 13:1–8. 10.1186/1471-2288-13-117 24047204 PMC3848812

[36] Smith J Firth J. Qualitative data analysis: the framework approach. Nurse Res. (2011) 18:52–62. 10.7748/nr2011.01.18.2.52.c8284 21319484

[37] Laverack G. Improving health outcomes through community empowerment: a review of the literature. J Health Popul Nutr. (2006) 24:113–20. 16796158

[38] Pope C Ziebland S May N. Analysing qualitative data. Brit Med J. (2000) 320:114–6. 10.1136/bmj.320.7227.114 10625273 PMC1117368

[39] Rappaport J. Studies in empowerment: introduction to the issue. Prev Human Serv. (1984) 3:1–7. 10.1300/J293v03n02_02

[40] Willis CD Riley BL Stockton L Abramowicz A Zummach D Wong G. Scaling up complex interventions: insights from a realist synthesis. Health Res Policy Syst. (2016) 14:88. 10.1186/s12961-016-0158-4 27993138 PMC5168709

[41] Onyegbule P Iyiegbuniwe E Sarter B James KS. Evidence-based intervention program for reducing obesity among African-American women in Southern California. Public Health Nurs. (2021) 38:350–6. 10.1111/phn.12830 33496008

[42] Malaijerdi Z Joveini H Hashemian M Borghabani R Maheri M Rohban A. Effects of an empowerment program for promoting physical activity in middle-aged women: an application of the health action process approach. Sport Sci Health. (2019) 15:595–603. 10.1007/s11332-019-00558-w

[43] Manavi N Abedi H. Investigating the effect of an empowerment program on physical activity of the elderly in Rezaeian health center, Iran, in 2014. Iran J Nurs Midwifery Res. (2016) 21:345–50. 10.4103/1735-9066.185570 27563315 PMC4979255

[44] Avery H Forss KS Ramgard M. Empowering communities with health promotion labs: result from a CBPR programme in Malmo, Sweden. Health Promot Int. (2021) 37:1–15. 10.1093/heapro/daab069 34263320 PMC8851348

[45] Dalsmo IE Haraldstad K Johannessen B Hovland OJ Chiduo MG Fegran L. Now I feel that I can achieve something: young tanzanian women's experiences of empowerment by participating in health promotion campaigns. Int J Environ Res Public Health. (2021) 18:8747. 10.3390/ijerph18168747 34444496 PMC8392774

[46] Fröberg A Jonsson L Berg C Lindgren EC Korp P Lindwall M. Effects of an empowerment-based health-promotion school intervention on physical activity and sedentary time among adolescents in a multicultural area. Int J Environ Res Public Health. (2018) 15:2542. 10.3390/ijerph15112542 30428548 PMC6267499

[47] Kobeissi L Nakkash R Ghantous Z Saad MA Yassin N. Evaluating a community based participatory approach to research with disadvantaged women in the southern suburbs of Beirut. J Community Health. (2011) 36:741–7. 10.1007/s10900-011-9368-4 21311960

[48] Israel BA Parker EA Rowe Z Salvatore A Minkler M Lopez J. Community-based participatory research: lessons learned from the centers for children's environmental health and disease prevention research. Environ Health Perspect. (2005) 113:1463–71. 10.1289/ehp.7675 16203263 PMC1281296

[49] Rutten A Abu-Omar K Frahsa A Morgan A. Assets for policy making in health promotion: overcoming political barriers inhibiting women in difficult life situations to access sport facilities. Soc Sci Med. (2009) 69:1667–73. 10.1016/j.socscimed.2009.09.012 19800160

[50] Brandstetter S McCool M Wise M Loss J. Australian health promotion practitioners' perceptions on evaluation of empowerment and participation. Health Promot Int. (2012) 29:70–80. 10.1093/heapro/das046 22987842

[51] Loss J. The empowerment concept: fuzzy, uncomfortable, uncertain–and indispensable. Gesundheitswesen. (2008) 70:713–4. 10.1055/s-0028-1102958 19085665

[52] Lindacher V Curbach J Warrelmann B Brandstetter S Loss J. Evaluation of empowerment in health promotion interventions: a systematic review. Eval Health Pro. (2017) 2017:1–42. 10.1177/0163278716688065 29172696

[53] Gibson CH. A concept analysis of empowerment. J Adv Nurs. (1991) 16:354–61. 10.1111/j.1365-2648.1991.tb01660.x 2037742

[54] Frahsa A Rutten A Abu-Omar K Wolff A. Movement as investement for health. Integrated evaluation in participatory physical activity promotion among women in dirfficult life situations. Global Health Prom. (2011) 18:31–3. 10.1177/1757975910393168 21721298

[55] Zimmerman M. Psychological empowerment: issues and illustrations. Am J Comm Psychol. (1995) 23:581–99. 10.1007/BF02506983 8851341

