# Corrigendum: “For me, it's just a piece of freedom”—Increased empowerment through physical activity promotion among socially disadvantaged women

**DOI:** 10.3389/fpubh.2023.1146584

**Published:** 2023-02-14

**Authors:** Alexandra Sauter, Annika Herbert-Maul, Karim Abu-Omar, Ansgar Thiel, Heiko Ziemainz, Annika Frahsa, Stephanie Linder, Anne Herrmann-Johns

**Affiliations:** ^1^Department of Epidemiology and Preventive Medicine, Medical Sociology, University of Regensburg, Regensburg, Germany; ^2^Department of Sport Science and Sport, Friedrich-Alexander-University Erlangen-Nuremberg, Erlangen, Germany; ^3^Institute of Sports Science, Eberhard Karls University of Tübingen, Tübingen, Germany; ^4^Institute of Social and Preventive Medicine, University of Bern, Bern, Switzerland

**Keywords:** physical activity, empowerment, community-based participatory research, women's health, low socioeconomic status, ethnic minority, qualitative research, health promotion

In the original published article, a data reanalysis is included which relates to a physical activity promotion project that has been running at various sites in Germany since 2005. The qualitative data used for the published reanalysis was collected between 2007 and 2011. A detailed description of the data set used and the reanalysis we conducted can be found in the methods section of the published article. In the published article, Roger, Rutten, Frahsa, Abu-Omar and Morga (2011) was cited three times. The corrigendum now includes further references to the work of Roger et al. (2011) and new to Rutten et al. (2008) in the introduction, method, and discussion sections of our paper.

A correction has been made to **Introduction**, [Paragraph 5]. These sentences previously stated:

“Several studies have examined the effects of the BIG approach on women's empowerment using data from the first BIG community (25, 26). This study builds on existing evidence by employing a more comprehensive dataset from more BIG communities in rural and urban areas with different population sizes.”

The corrected sentences appear below:

“Previous work from Rütten et al. in 2008 (25) and Röger et al. in 2011 (26) have examined the effects of the BIG approach on women's individual, organizational and community empowerment using data from the first BIG community (25, 26). This study builds on existing evidence from these and other previous works. It employing a more comprehensive dataset from more BIG communities in rural and urban areas with different population sizes. To answer the research question, theoretical empowerment approaches used in previous BIG-studies have been employed (25, 26).”

A correction has been made to **Methods**, “*Design*,” [Paragraph 1]. This sentence previously stated:

“For the comprehensive data analysis, we used 53 interview transcripts from five BIG communities, with a total of 63 interviewees (see Table 1).”

The corrected sentence appears below:

“For the comprehensive data analysis, we used 53 interview transcripts from five BIG communities [including interviews from pilot community A, see also Röger et al. 2011 (26) and Rütten et al. 2008, (25)], with a total of 63 interviewees (see Table 1).”

A correction has been made to **Discussion**, “*Principal findings*,” [Paragraph 1]. This sentence previously stated:

“Confirming existing evidence (26), our findings show across all five communities that women who only participated in PA courses mainly had effects on an individual empowerment level.”

The corrected sentence appears below:

“Our findings could confirm existing evidence from Rütten et al. (25) and Röger et al. (26). By using a larger set of data from different communities, we could show that some of the effects of the BIG pro-ject are not exclusive for pilot community A [see also Rütten et al. (25) and Röger et al. (26)], but could also be found when transferring the project to other sites.”

A correction has been made to **Discussion**, “*Principal findings*,” [Paragraph 1]. This sentence previously stated:

“This skill development even helped some back into the workforce.”

The corrected sentence appears below:

“This skill development even helped some back into the workforce (25, 26).”

A correction has been made to **Discussion**, “*Strength and Limitations*,” [Paragraph 1]. These sentences previously stated:

“Our in-depth and rich data material shows how the provision of need-based PA offerings and the dynamics of interactions between the participants strengthen the women's individual empowerment in terms of self-efficacy, competencies, power, and social capital (e.g., advancing social networks, bonding with other women, experiencing group solidarity, and belongingness).”

The corrected sentences appear below:

“We conducted a secondary analysis of existing data (25, 26) which was supplemented and updated by additional interview data to answer the research question underpinning this current study. Due to this in-depth and rich data material shows how the provision of need-based PA offerings and the dynamics of interactions between the participants strengthen the women's individual empowerment in terms of self-efficacy, competencies, power, and social capital (e.g., advancing social networks, bonding with other women, experiencing group solidarity, and belongingness).”

The authors apologize for this error and state that this does not change the scientific conclusions of the article in any way. The original article has been updated.

